# Carbon allocation during defoliation: testing a defense-growth trade-off in balsam fir

**DOI:** 10.3389/fpls.2015.00338

**Published:** 2015-05-13

**Authors:** Annie Deslauriers, Laurie Caron, Sergio Rossi

**Affiliations:** Département des Sciences Fondamentales, Université du Québec à Chicoutimi, Chicoutimi, QCCanada

**Keywords:** spruce budworm outbreak, terpenoids, carbon allocation, starch, trade-off, wood formation

## Abstract

During repetitive defoliation events, carbon can become limiting for trees. To maintain growth and survival, the resources have to be shared more efficiently, which could result in a trade-off between the different physiological processes of a plant. The objective of this study was to assess the effect of defoliation in carbon allocation of balsam fir [*Abies balsamea* (L.) Mill.] to test the presence of a trade-off between allocation to growth, carbon storage, and defense. Three defoliation intensities [control (C-trees, 0% defoliation), moderately (M-trees, 41–60%), and heavily (H-trees, 61–80%) defoliated] were selected in order to monitor several variables related to stem growth (wood formation in xylem), carbon storage in stem and needle (non-structural soluble sugars and starch), and defense components in needles (terpenoids compound) from May to October 2011. The concentration of starch was drastically reduced in both wood and leaves of H-trees with a quasi-absence of carbon partitioning to storage in early summer. Fewer kinds of monoterpenes and sesquiterpenes were formed with an increasing level of defoliation indicating a lower carbon allocation for the production of defense. The carbon allocation to wood formation gradually reduced at increasing defoliation intensities, with a lower growth rate and fewer tracheids resulting in a reduced carbon sequestration in cell walls. The hypothesis of a trade-off between the allocations to defense components and to non-structural (NCS) and structural (growth) carbon was rejected as most of the measured variables decreased with increasing defoliation. The starch amount was highly indicative of the tree carbon status at different defoliation intensity and future research should focus on the mechanism of starch utilization for survival and growth following an outbreak.

## Introduction

In eastern North America, the spruce budworm (*Choristoneura fumiferana* Clem.) population is undergoing an explosion, and the defoliated area in Quebec has doubled every year since 2005, exceeding 3 M ha in 2014 (Direction De La Protection Des Forêts, 2014). Spruce budworm is one of the major natural disturbances of boreal forest (Fleming et al., 2002), causing dramatic growth reductions and stand mortality with future outbreaks predicted to last 6 years more and to produce 15% greater defoliation (Gray, 2008). Balsam fir [*Abies balsamea* L. (Mill.)] is the preferred host following by white spruce [*Picea glauca*, (Moench) *Voss*] and black spruce [*Picea mariana B.P.S.* (Mill.)]. Because of average mortality around 50% (Bergeron et al., 1995) and volume losses varying between 32 and 48% (Ostaff and Maclean, 1995), outbreaks play a significant role in the carbon (C) flux of the forests in Quebec, with 2.87t C ha^-1^ year^-1^ of losses being measured in defoliated plots (Zhang et al., 2014). At the tree level, a dramatic decrease in leaf biomass (i.e., reduction in C source) is expected to affect the C allocation priorities in growth, storage, and defense components (Koricheva et al., 1998).

The strategy of C use and accumulation reflects plant ability to withstand defoliation (Vanderklein and Reich, 1999). Compared with broadleaves, evergreen trees are known to store a lower proportion of C in wood than in leaves (Hoch et al., 2003; Fajardo et al., 2013). However, this strategy makes evergreen trees more prone to carbon depletion and eventually to mortality under prolonged defoliations (Piper and Fajardo, 2014). The remaining leaves provide energy only to maintain metabolism and growth of the subsequent leaves (Li et al., 2002) while stem radial growth slows down or stops after a few years of defoliation (Krause and Morin, 1995a; Rossi et al., 2009). According to Vanderklein and Reich (1999), any change in the C-balance of defoliated trees should be noted first in the starch pool. Defoliation thus alters the non-structural soluble carbohydrates (NSCs) contained in most tree compartments (i.e., stem, leaves, and roots) by reducing the amount of reserves (Myers and Kitajima, 2007), especially starch (Ericsson et al., 1980; Hudgeons et al., 2007; Jacquet et al., 2014). According to Atkinson et al. (2014), the mechanism of utilization of *stored resources following defoliation* is not completely understood, thus leaving a gap in the knowledge on characterization of the effect of a carbon decrease on the other sink activities (i.e., growth, metabolism, and defense).

Although the stem growth reductions caused by spruce budworm outbreaks are well known (Blais, 1961, 1983; Bergeron et al., 1995; Krause and Morin, 1995b), the intra-annual dynamics of xylem formation in defoliated trees has never been assessed, except under artificial conditions (Rossi et al., 2009). Wood formation in the stem requires several C-compounds (Simard et al., 2013; Deslauriers et al., 2014) mainly from newly synthesized NSCs (Hansen and Beck, 1990, 1994). A reduced carbon allocation to radial growth is thus expected under defoliation (Vanderklein and Reich, 1999; Jacquet et al., 2014) because of its lower priority in respect to other sinks of plants, as also demonstrated by the missing rings after several years of defoliation (Krause and Morin, 1995a).

Needles of conifers contain secondary metabolites with defense functions (Schonwitz et al., 1991; Carlow et al., 2006), of which monoterpenes are the dominant ones in resin (Trowbridge et al., 2014). Their mechanisms of production are still poorly understood because several factors such as available nutrients (Lamontagne et al., 2000, 2002), water, C-reserve (Trowbridge et al., 2014), and light (Niinemets et al., 2004) influence their production. The concentration of monoterpenes can also be affected by the emission of volatile compounds (Trowbridge et al., 2014) and their physicochemical characteristics such as volatility, solubility, and diffusivity (Niinemets et al., 2004; Niinemets, 2010; Blanch et al., 2011). Many studies have shown that the concentration and emission of monoterpenes in needles can be affected by herbivore and insect damage (Lamontagne et al., 2000; Miller et al., 2005; Carlow et al., 2006; Holopainen and Gershenzon, 2010). According to Holopainen and Gershenzon (2010), the increase in monoterpene biosynthesis and emission by insect damage has two causes: breakage of structures in which volatiles are stored (resin ducts in the case of conifers) and stimulated production of compounds in response to the enzymes or peptides inoculated by the insects feeding on needles.

Defoliation is one of the most problematic events because the loss in carbon production can rapidly become limiting for growth, storage, and defense. Two major hypotheses regarding carbon allocation to growth, storage, and defense compounds have been proposed: the Growth-Differentiation Balance Hypothesis (GDBH) and the Carbon and Nutrient Balance (CNB) hypothesis (see reviews by Herms and Mattson, 1992 and Koricheva et al., 1998). These hypotheses aim at predicting the possible trade-off in carbon allocation between growth and defense, in the case of growth limited by an environmental factor (GDBH) or either carbon or nutrients (CNB; Koricheva et al., 1998). In the case of a carbon limitation, such as during defoliation, both hypotheses predict a decrease in the carbon-based secondary metabolism (terpenoids) and growth. In defoliated balsam fir, these allocation patterns and possible trade-offs still remain unexplored. We therefore took advantage of the current epidemic in Quebec (Canada) to investigate the integrative carbon allocation response to different defoliation intensity. Monitoring defoliated mature trees in the field allowed to adequately investigate the long term effects of canopy defoliation on growth and reserve.

**FIGURE 1:**
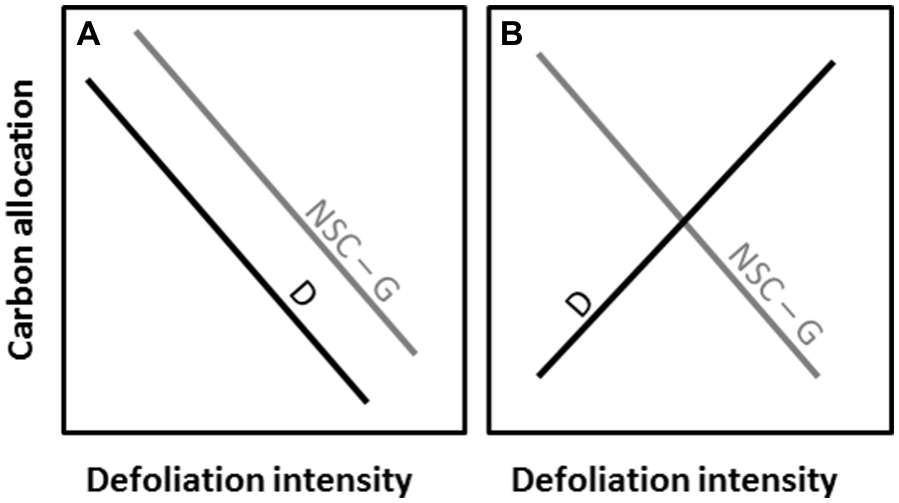
**Theoretical patterns of carbon allocation to defense (D), non-structural carbon (NSC) and structural growth (G) with increasing defoliation intensity**. The patterns are illustrated with the absence **(A)** or presence **(B)** of trade-off between the carbon allocations.

The objective of the study was to assess the effect of defoliation intensity on carbon allocation of balsam fir [*A. balsamea* (L.) Mill.] by testing two divergent theoretical patterns underlying the absence (**Figure 1A**) or presence (**Figure 1B**) of trade-off between the allocations to defense components and non-structural (NCS) and structural (radial growth) carbon at different defoliation intensities (**Figure 1**).

## Materials and Methods

### Study Sites

The study was performed in the Laurentide Wildlife Reserve of Quebec, Canada. Two defoliated sites, classified as moderately (M, 41–60%) and heavily (H, 61–80%) defoliated were selected according to Direction De La Protection Des Forêts (2011). A control site, showing no sign of defoliation was located at about 1 km from the defoliated sites. Six dominant or co-dominant balsam fir trees were randomly selected in each site (**Table 1**). Trees had comparable height and diameter (**Table 1**), indicating a similar development stage.

**Table 1:** Diameter (DBH 1.3 m) and average height of the six sampled trees in each study sites.

Study trees	Geographic coordinates	Altitude (m)	Diameter (cm)	Height (m)
C-tree	48° 12′ 43.5′ N, 71° 14′ 26.6′ W	370	18.97 ± 2.31	16.40 ± 1.55
M-tree	48° 16′ 06.4′ N, 71° 14′′ 29.6′ W	290	17.08 ± 1.67	14.28 ± 0.73
H-tree	48° 16′ 06.0′ N, 71° 14′′ 52.5′ W	320	15.42 ± 2.60	13.57 ± 2.12

### Sample Collection

The sampling was performed from May to October 2011 in order to follow wood formation and the variation in sugar, starch, and defense components during the growing season. Samples from each tree were collected every 2 weeks starting in spring [Day Of the Year (DOY) 139] except during the period of heaviest defoliation in June, when sampling was done weekly. Wood microcores were collected following a spiral trajectory on the stem from 30 cm below to 30 cm above breast height (1.3 m) using Trephor (Rossi et al., 2006a). Short canopy branches were collected in the middle of the canopy by using a telescopic branch pruner. From this sample, a twig was used to assess the relative water content (RWC) and the needles were kept to measure leaf chemistry parameters (soluble sugar, starch, and defense components). The tree stems were cored about once per month, with an increment borer, to measure the starch and sugar contents in the outermost layer (approximately the first 5 cm) of xylem.

### Terpenoid Compounds

Needles of 1–3 years were sampled at about 8 m in height (mid-canopy), placed in paper bags and stored at -20°C. The needles were immersed in liquid nitrogen at -196°C to stop all enzymatic activities and 5 g of needle was ground (Retsch MM200 Vibrant) for 5 min at 1 μm and stored at -20°C. The volatile compounds were analyzed by static headspace gas chromatography (HS-GC) according to Caron et al. (2013).

### Stem Relative Water Content (RWC)

Twigs were immediately weighed to obtain fresh mass (g) and placed in the dark at 4°C in a transparent bottle containing distilled water. After 24 h, the twigs were weighed to obtain the rehydrated mass (g) and placed for 48 h in an oven at 80°C. Samples were weighed again to obtain the dry mass (g) (Giovannelli et al., 2007). The RWC was calculated as:

$$RWC=\frac{M_{f}-M_{d}}{M_{r}-M_{d}}\times 100$$

where *M_f_* is the fresh mass, *M_r_* the rehydrated mass, and *M_d_* the dry mass.

### Total Soluble Sugars and Starch

The needles and wood cores collected during sampling were placed in paper bags and stored at -20°C for the analysis of soluble sugar and starch. Later, samples were immersed in liquid nitrogen at -196°C to stop all enzymatic activities and dehydration was performed by lyophilisation for a period of 5 days (Deslauriers et al., 2009). needles and wood were ground (Retsch MM200 Vibrant) for 5 min at 1 μm. Samples were then stored at -20°C until analysis.

The analyses of total soluble sugars (TSS) and starch were done according to (Chow and Landhäusser, 2004). This method extracts the sugars with hot ethanol (80%) and analyses them using the phenol-sulfuric acid technique. The absorbance of the samples was measured at 490 nm with a UV–VIS spectrophotometer. The concentration of soluble sugars was converted to mg per g of dry weight (mg/g_dw_). The remaining pellets were used for the determination of starch by using a technique of enzymatic digestion with amylase and amyloglucosidase enzymes. Glucose chains forming the starch are split and the concentration of these compounds are found by determining their absorbance at 520 nm with a UV–VIS spectrophotometer. Starch quantities are then converted to mg/g_dw_.

### Stem Growth and Anatomy

The microcores were placed in Eppendorf microtubes in a 10% ethanol solution and stored at 5°C to avoid tissue deterioration. Microcores were fixed in paraffin with successive immersions in ethanol and D-limonene and embedded in paraffin in a circulator (Leica TP1020). Sections of about 7 μm were cut with a rotary microtome (Leica RM 2245) and fixed on slides with albumin. The sections were stained with a 0.05% solution of cresyl violet acetate and examined within 10–25 min under visible and polarized light at magnifications of 400–500 × to differentiate the developing and mature xylem cells along three lines (Deslauriers et al., 2003; Rossi et al., 2006a).

The sections collected during the last sampling were stained with safranin and permanently fixed on slides with Permont for the analysis of cell size. Numerical images of the tree-ring were taken at a magnification of 200× with a camera mounted on the microscope (Deslauriers et al., 2003). Cell length, lumen, area, and cell wall thickness (all in micron, μm) were measured along three radial files per section with Wincell^TM^ (Regent Instrument) and averaged for each tree. The width of the tree-rings produced during 2007–2011 was also measured on the sections to the nearest 0.01 mm.

### Data Analyses

Different approaches were used to verify the effect of defoliation intensity on carbon allocation in the trees: First, the effect of defoliation intensity on defense components and non-structural (NCS) and structural (growth) carbon was verified separately for each category of variable; Second, the trade-off was verify by using a set of quantitative variables in one analysis (Canonical discriminant analysis) to identify which one affect the separation between defoliation levels.

#### Separate Effect of Defoliation Intensity on Growth, Carbon, and Defense

The effect of defoliation on the previously formed tree-ring was illustrated by calculating an index of tree-ring width (TRW). This index was calculated for each year *n* based on the year 2007 (beginning of the spruce budworm defoliation) according to the following formula:

$$Tree-ring\,\;\;index=\frac{{TRW}_{n}}{{TRW}_{2007}}-\;1$$

The effect of defoliation on intra-annual growth was tested by a model fitting approach (Potvin et al., 1990; Giovannelli et al., 2007) using Gompertz function. Gompertz functions were fitted by non-linear regressions (NLIN procedure in SAS) to estimate the pattern of intra-annual growth (y) against time (t, in DOY; Rossi et al., 2006b):

$$y=A\,\;\;\;\exp-\left(e^{\left(\beta -kt\right)}\right)$$

where the parameters *A*, β, and κ are the growth asymptotes, time axis placement and rate of change of the curve, respectively. The rate of growth (*r*, number of cell.day^-1^) was calculated as *r* = *A*κ/4.

The presence of terpenoids was compared between the defoliation classes using Fisher exact tests and contrasts, where the response variable was implemented as binomial variable representing the presence (1) or the absence (0) of each molecule. The problem of multiple testing, which arises when performing many hypothesis tests on the same data set, was resolved by excluding the probability of declaring false significances by adjusting the *P*-values using 10,000 bootstrap resampling with replacement [PROC MULTTEST, SAS version 9.4 (SAS Institute, Cary, NC, USA)]. For the remaining variables (RWC, soluble sugars and starch in leaves and wood, β-phellandrene, δ-3-carene), the effect of defoliation was tested by Mixed Models (MIXED procedure in SAS) using a factorial model with tree (df = 6) as the random term for testing the fixed effects [defoliation (Def), DOY, and their cross effect]. The Mixed Models were used to take into account the repeated measurements taken on the same trees along the growing season (longitudinal data) as these were correlated.

#### Trade-off

The trade-off (**Figure 1**) between the allocations to defense components and non-structural (NCS) and structural (growth) carbon was tested with Canonical discriminant analysis (CANDISC procedure in SAS, SAS Institute Inc., Cary, NC, USA). The variables used to investigate the effect of the defoliation intensity in the Canonical discriminant analysis were RWC, TSS and starch (wood and leaves), β-phellandrene, δ-3-carene, and the number of monoterpenes and sesquiterpenes, total number of cells (*A*) and the rate of growth (cell.day^-1^). Canonical discriminant analysis finds linear combinations of quantitative variables that provide maximal separation between classes (i.e., defoliation intensity) and successively tests the hypothesis that the class means are equal in the population. Wilks’ and Mahalanobis squared distance were performed for overall and multiple comparisons of the class means, respectively.

The percentage of difference between values of C-trees and M- or H-trees was calculated as:

$$Percent\,\;\;difference=100\times \frac{\left|v_{1}-v_{2}\right|}{(v_{1}-v_{2})/2}$$

where ν_1_ represents any given measured value of C-trees and ν_2_ represents any given measured value of M- or H-trees.

## Results

### Allocation to Structural Growth at Different Defoliation Intensity

**FIGURE 2:**
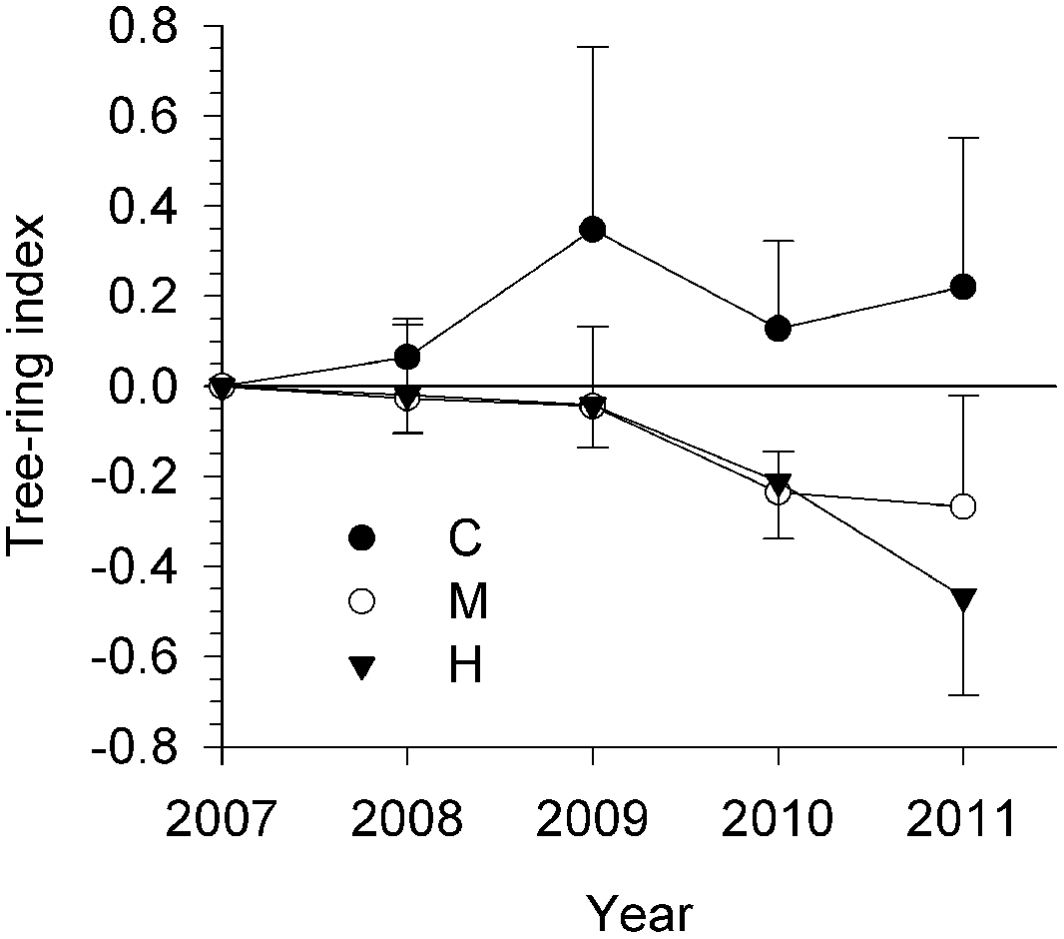
**Measurement of growth rings in the 5 years up to 2011 for the three study sites (C, control trees; M and H are moderately and heavily defoliated trees)**. Vertical bars represent the SD calculated among six trees.

Stem growth of the defoliation intensity groups had diverging patterns (**Figure 2**). From 2008 to 2011, C-trees showed tree-rings with positive index, thus increasing in width. However, tree-ring indexes in defoliated trees were lower than 0 and gradually decreasing. M- and H- trees exhibited analogous values until 2010. In 2011, the tree-ring index seemed to have attained a plateau at -0.27 in M-trees, while it decreased again in H-trees, attaining -0.47.

The seasonal dynamics of xylem cell production were similar between defoliation intensities, with the first cells appearing during the same sampling week at the beginning of June (**Figure 2**). An abrupt increase in the number of cells then occurred, but lasting longer in C-trees and finishing in September. Smaller increases in cell number were observed in defoliated trees, which resulted in less cells produced along the tree-ring (**Table 2**, parameter *A*). The model detected a significant effect of defoliation between the radial growth curves (*F* = 36.67, *P* < 0.001, **Table 2**). M- and H-trees exhibited a marked reduction in the growth rate, with values of 0.26 and 0.17 cell.day^-1^, respectively, compared with 0.56 cell.day^-1^ in C-trees. Thus, the rate (*r*) and total cell production (*A*) in H-trees were reduced by more than half with respect to C-trees.

**Table 2:** Growth fitting and comparisons among defoliation category (control, moderately, and heavily defoliated).

Parameter	C-tree	M-tree	H-tree
*A*	54.16 2.64	34.33 1.72	21.191.60
β	7.23 1.66	5.14 0.98	5.79 1.68
κ(10)	4.20 0.97	3.07 0.60	3.32 0.99
*r*	0.56	0.26	0.17

**Statistics**	**Growth curve fitting**	**Comparison of defoliation level**

df (*v*_1_,-*v*_2_)	3, 81	3, 86	3, 86	8, 250
*F*-value	252.24	337.77	136.72	36.67
*P*-value	0.001	<0.001	0.001	0.001

*A, β, and κ represent the fitted parameters of the Gompertz function, *r* indicates a weighted mean absolute rate (*r*, cell.da^-1^). Each *F*- and *P*-values are presented for growth response curves, fitted to the number of cells. An *F*-value was calculated among defoliation levels to compare non-linear tree-ring growth (see Methods).*

**FIGURE 3:**
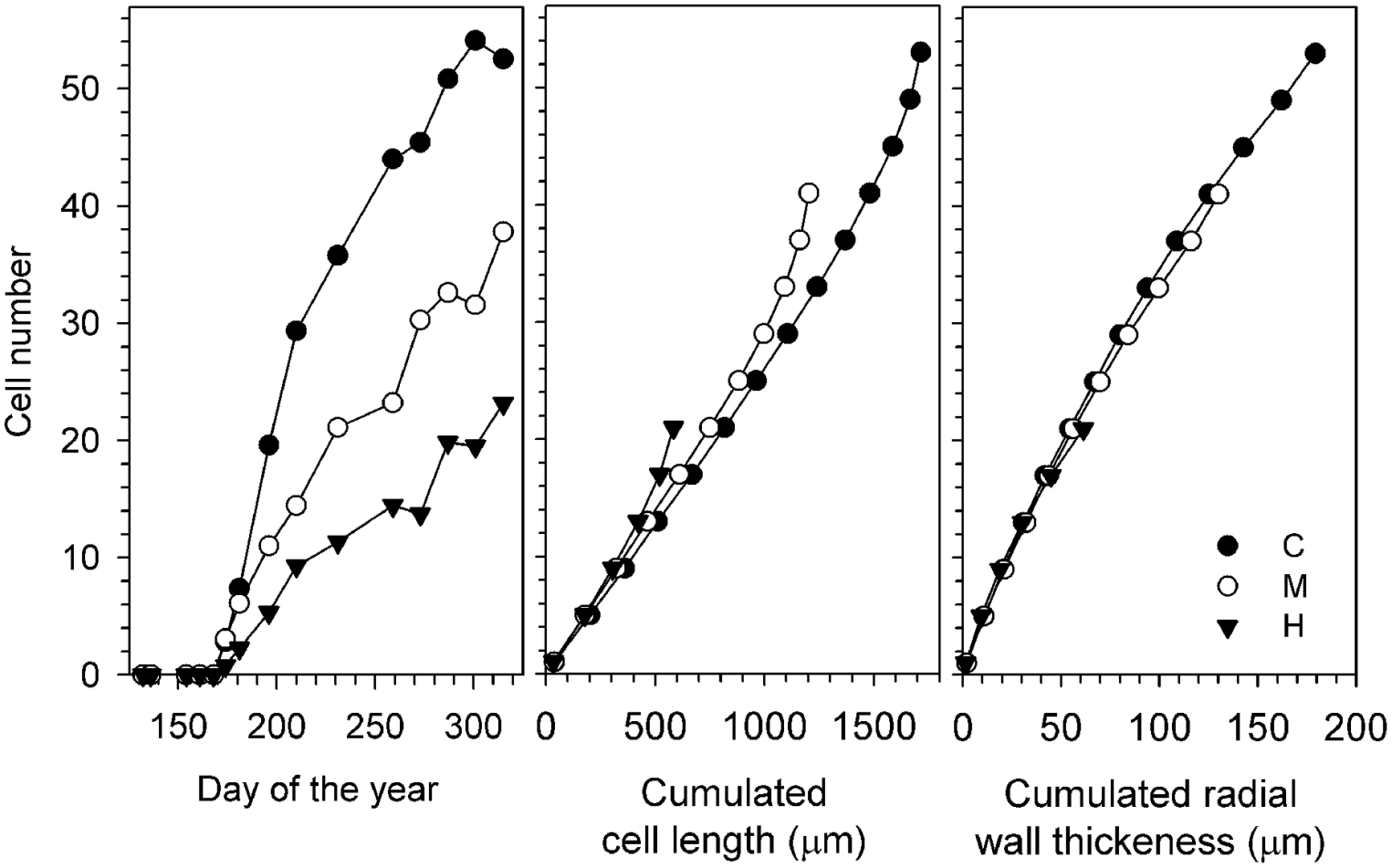
**Wood formation during the 2011 growing season for the three study sites: increase in the number of cells **(Left)** and anatomical component represented by cumulated cell length (**Center**, μm) and cumulated radial wall thickness (**Right**, μm)**. H, heavily defoliated trees; M, moderately defoliated trees; C, control trees.

On average, the tree ring of C-trees was 1714 μm, calculated by cumulating the radial length of each xylem cell (**Figure 3**). The growth reduction of defoliated trees resulted in fewer xylem cells and thinner tree rings. The reduction in thickness was related to the degree of defoliation, with M- and H-trees showing a cumulated cell length of 1203 and 583 μm, respectively. Similarly, also the investment in wall material of defoliated trees was reduced, mainly because of the fewer cells (**Figure 3**). In radial direction, C-trees showed a cumulated wall thickness of 179 μm, while M- and H-trees reached only 130 and 61 μm, respectively.

### Twigs Water Content

**FIGURE 4:**
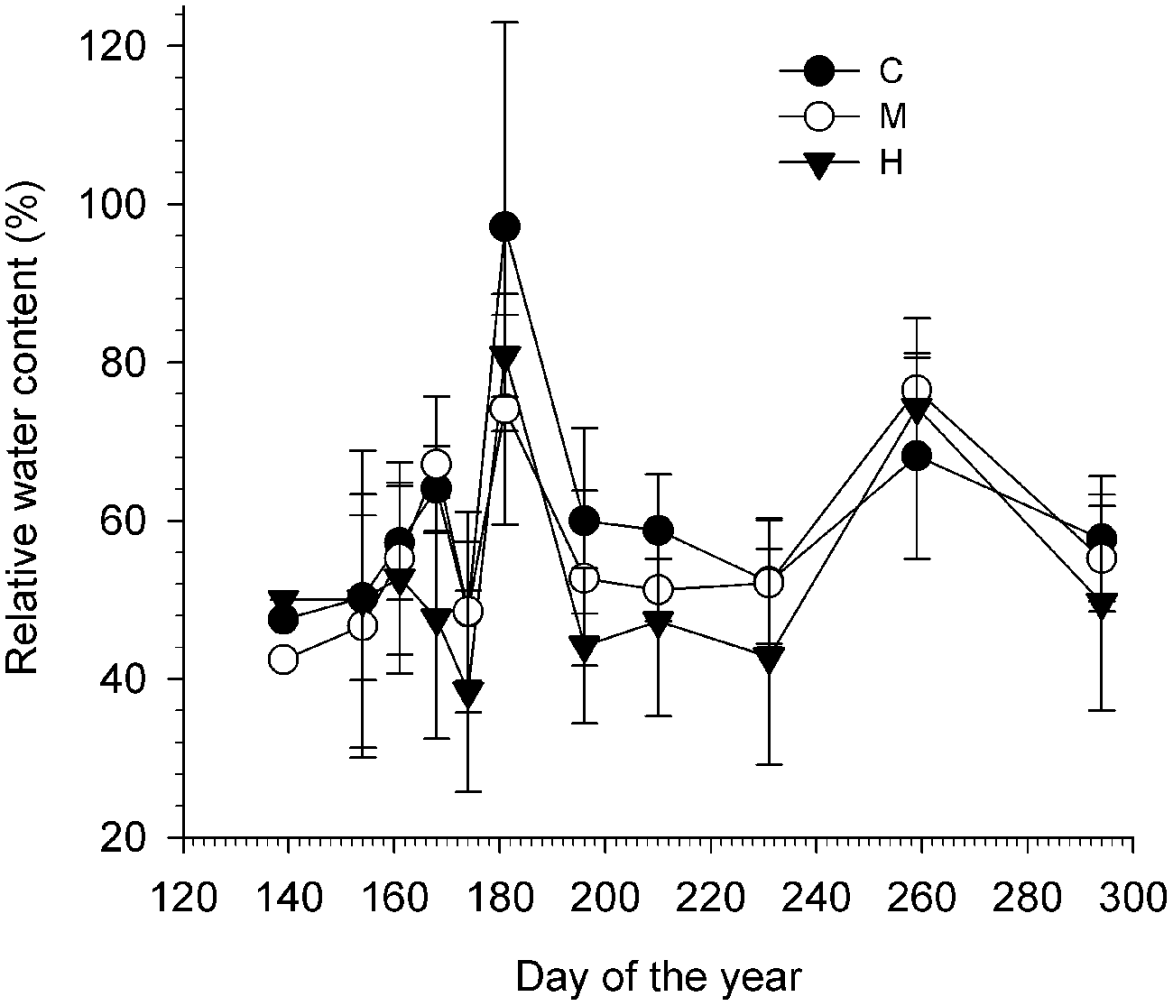
**Relative water content (RWC, %) of the twigs calculated for each defoliation class (C, control trees; M and H are moderately and heavily defoliated trees) along the growing season**. Vertical bars represent the SD calculated among six trees.

Defoliation slightly decreased the water content in the twigs as a lower RWC was measured throughout the growing season on the heavily defoliated trees (**Figure 4**; **Table 3**). In general, the mean RWC of C- and M-trees varied between 56 and 60%, which were both significantly higher than H-trees (mean of 52%). RWC was not constant during the growing season (significant effect of DOY, *P* < 0.001) and varied according to the precipitations. A cross effect was also found (*P* < 0.05), indicating that at some sampling dates, H- trees had higher RWC (**Table 3**).

**Table 3:** Mean seasonal values and effect of defoliation class on the measured variables in the leaf and wood (C-, M-, and H-tree are control, moderately and heavily defoliated trees).

		Mean values			*F*-value		
Categories	Variable	C-tree	M-tree	H-tree	Defoliation	DOY	DOY × Defoliation
Water	RWC	60.14 ± 17.09	56.55 ± 14.17	52.55 ± 16.69	8.99***	24.13****	1.76*
Carbon	TSS-L	47.87 ± 19.44	49.85 ± 17.82	50.96 ± 21.33	1.27	19.40****	9.78***
	TSS-W	5.31 ± 2.03	4.80 ± 1.52	5.71 ± 2.87	2.34	3.72***	3.20**
	Sta-L	63.93 ± 61.51	37.89 ± 42.51	21.02 ± 32.61	60.93****	50.90****	6.86****
	Sta-W	1.12 ± 1.25	0.55 ± 0.65	0.70 ± 1.03	6.03**	15.97****	0.94
Defense	β-phe	156.32 ± 180.14	236.43 ± 281.43	114.11 ± 157.81	11.96****	15.46****	3.21****
	δ-3*-*c	293.56 ± 304.36	229.69 ± 332.37	78.51 ± 214.66	13.37****	3.75***	1.67*

F-statistics and *P*-values for defoliation, days of the year (DOY) and their cross effect are reported for each variable. For all variables (except Sta-W and TSS-W), the degree of freedom (ν_1_,ν_2_) of the *F* tests were (2,165) for defoliation, (10,165) for DOY and (20,165) for their cross effect. For Sta-W and TSS-W, the degree of freedom were (2,90) for defoliation, (5,90) for DOY and (10,90) for their cross effect. RWC, relative water content of the twigs (%); TSS-L, total soluble sugar in the leaf (mg/g_*dw*_); Sta-L, starch content in the leaf; β-phe, β-phellandrene in the leaf (mg/g_*dw*_); δ-3*-*c, δ-3*-*carene (mg/g_*dw*_) in the leaf; TSS-W, total soluble sugar in the wood (mg/g_*dw*_); Sta-W, starch content in the wood (mg/g_*dw*_). *P < 0.05, **P < 0.01, ***P < 0.001, ****P < 0.0001.

### Allocation to Non-Structural Carbon in Leaves and Wood at Different Defoliation Intensity

**FIGURE 5:**
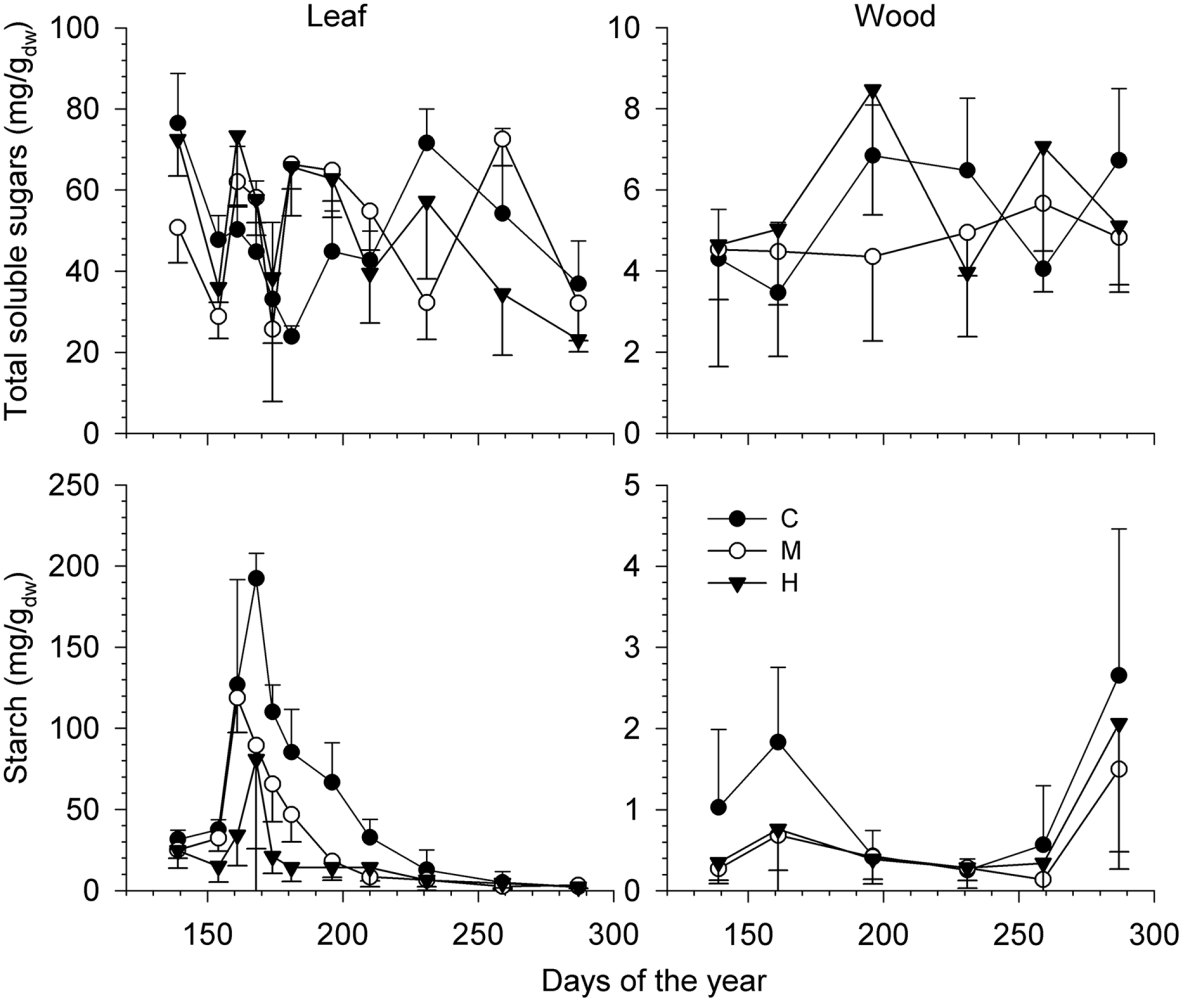
**Variation in the soluble sugars and starch (mg/g_dw_) measured in leaf **(Right)** and stem wood **(Left)** during the growing season for the different defoliation classes (C, control trees; M and H are moderately and heavily defoliated trees)**. Vertical bars represent the SD calculated among six trees. Note the different ranges of the vertical axes.

Total soluble sugars in leaves and stem wood varied without a clear seasonal pattern (**Figure 5**) and the intensity of defoliation did not affect the amount found during the sampling period (**Table 3**). Although having different meaning, the amount of TSS was higher in leaves compared with the xylem, with means around 50 and 5 mg/g, respectively. The amount of starch, however, was significantly affected by defoliation in both leaves (*P* < 0.0001) and wood (*P* < 0.01; **Table 3**). A distinct seasonal trend was also found in both organs during the growing season (effect of DOY, *P* < 0.0001). In leaves, the amount of starch decreased from DOY 161 to 196 with a more pronounced drop in M-trees and H-trees (**Figure 5**). After this, the variations were less pronounced until the end of the growing period. In the leaves of H-trees, the peak of starch mobilization only lasted 1 week (**Figure 5**). A marked difference was found in wood, but only during the starch mobilization in June (**Figure 5**) when C-trees had higher quantities than defoliated trees. At the end of the growing season (i.e., after DOY 280), starch mobilization was observed but only in the wood.

### Allocation to Terpenoids at Different Defoliation Intensity

**FIGURE 6:**
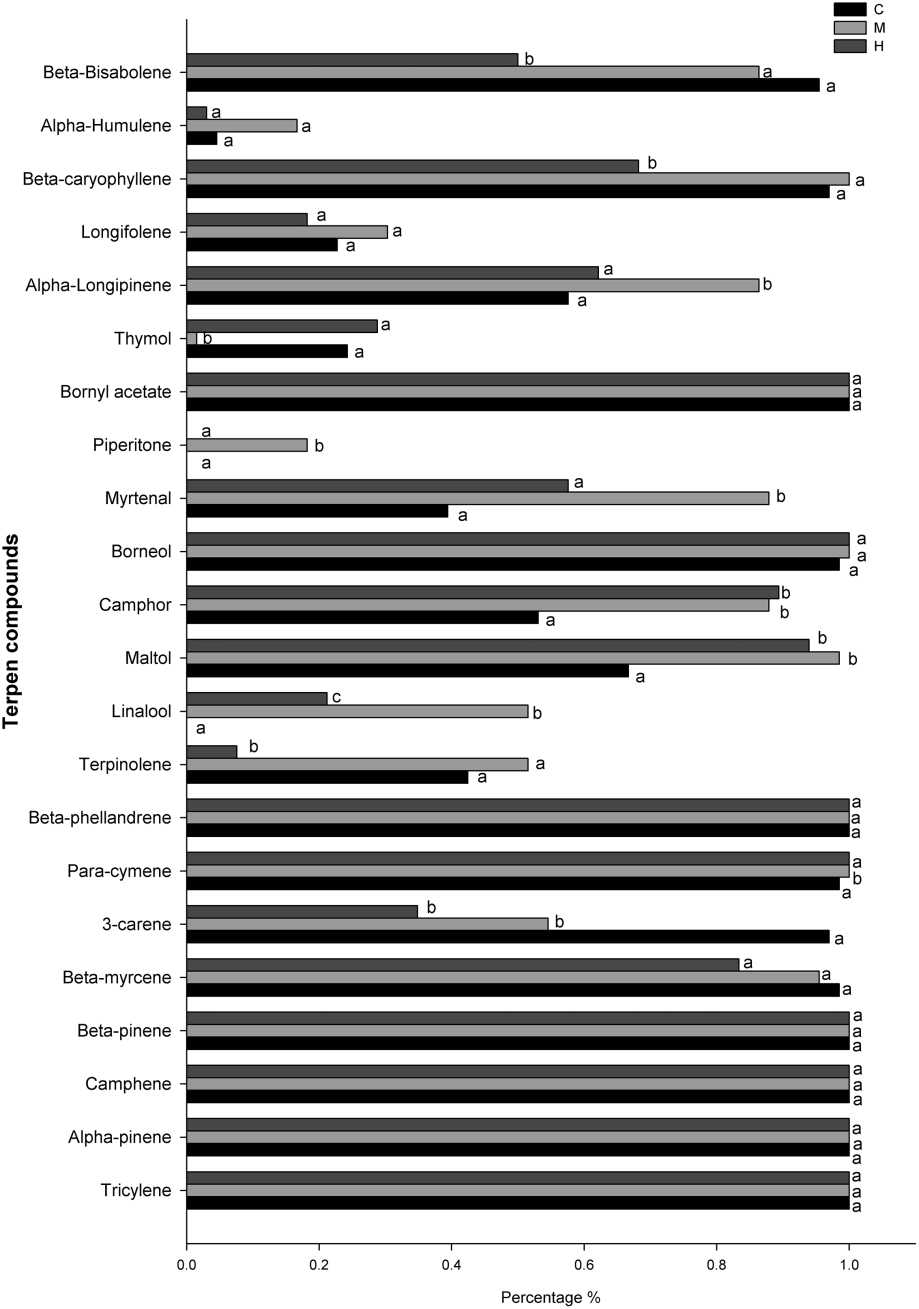
**Percentage of occurrence (presence) of defense compounds in the needle among all sampling dates for the different defoliation classes (C, control trees; M and H are moderately and heavily defoliated trees)**. Different letters (a, b, c) indicate a significant difference (*p* < 0.05) between defoliation intensity.

The most frequent volatile compounds found in needles (100% of presence during the growing season) were monoterpenes or sesquiterpenes: tricyclene, α-pinene, camphene, β-pinene, β-myrcene, borneol, bornyl acetate, with no significant difference found between defoliation intensities (**Figure 6**). Other, less frequent volatile molecules, such as longifolene (30% of presence) and α-humulene (10–20% of presence) also showed no significant difference between defoliation intensities. Some compounds were either found in higher (para-cymene, myrtenal, and piperitone) or lower (thymol) proportion in M-trees compared with C- and H-trees. In H-trees, a difference was observed only for three sesquiterpenes terpenes (β-bisabolene, β-caryophyllene, and terpinolene) because their frequency of occurrence was about 30% less compared with C- and M-trees. The δ-3-carene, maltol, and camphene showed similarities between the two defoliated sites with a higher percentage of presence compared with the control. One compound, linalool, was absent in the C-trees while its occurrence increased in defoliated trees with 21 and 51% in H- and M-trees, respectively (**Figure 6**).

**FIGURE 7:**
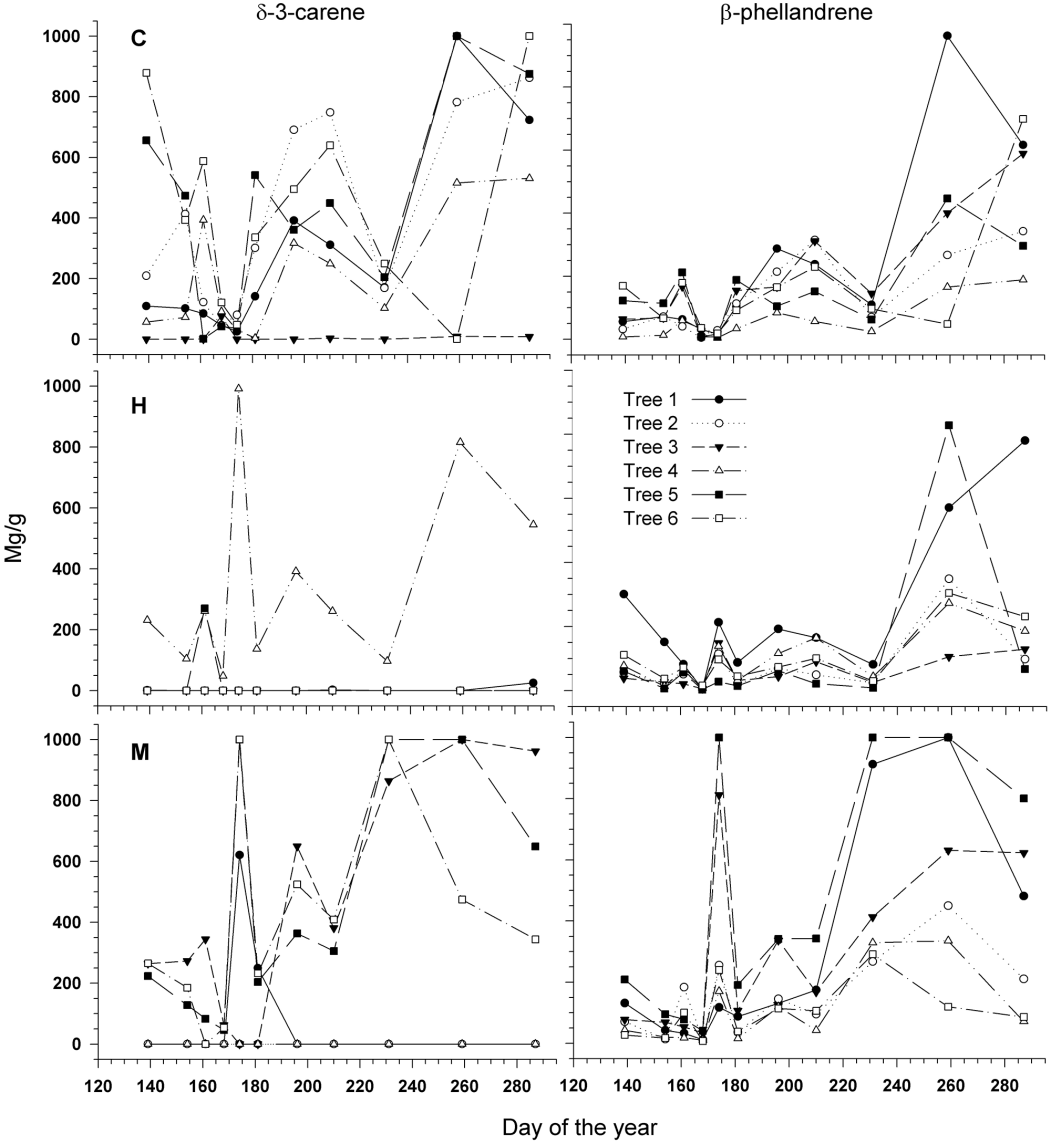
**Quantity (mg/g_dw_) of δ-3-carene **(Left)** and β-phellandrene **(Right)** measured during the growing season in six trees per defoliation intensity (C, control trees; M and H are moderately and heavily defoliated trees)**. All the trees (numbered from 1 to 6 for each intensity) are represented because of the big difference between trees, especially for heavily defoliated trees.

Two compounds, δ-3-carene and β-phellandrene, were selected for a quantitative analysis as marked differences were noted between the defoliation classes and date of sampling by observing the chromatograms. In **Figure 7**, each tree is represented by a single curve, as for the δ-3-carene for example, only one H-tree synthesized this molecule, creating difficulties in illustrating means and SD. The δ-3-carene concentration varied from 0 to 1000 mg/g with a similar range between defoliation intensities (**Figure 7**) and a distinct seasonal pattern. For many H- and M-trees, it was impossible to quantify the δ-3-carene, because concentrations were below the HS-GC detection limit. The quantity of β-phellandrene in each tree also followed a similar intra-annual variation among sites with values ranging from 0 to 960 mg/g (**Figure 7**). Significant differences were found between the defoliation classes for both compounds (*P* < 0.0001, **Table 3**). A lower amount of δ-3-carene was found in H-trees than in M- and C-trees. A higher amount of β-phellandrene was found in M-trees.

### Testing the Defense-Growth Trade-off

**FIGURE 8:**
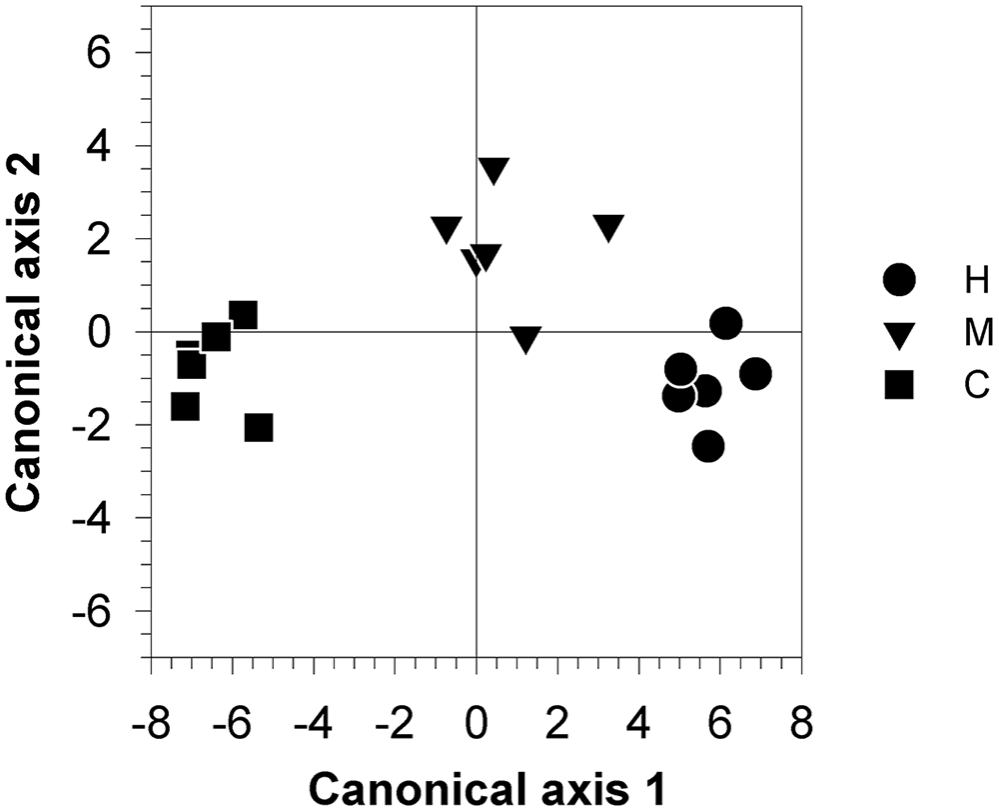
**Canonical discriminant analysis performed using variables representing the allocations to defense components and to non-structural (NCS) and structural (growth) carbon [RWC, total soluble sugars and starch (wood and leaves), β-phellandrene, δ-3-carene, and the number of monoterpenes and sesquiterpenes, total number of cells (**A**) and rate of growth (cell.day^-1^)] collected from 18 trees belonging to three different defoliation intensities (H, heavily defoliated trees; M, moderately defoliated trees; C, control trees)**.

Canonical axis 1 (Can 1) represented 93.3% of between-class variation and discriminated between the three groups (**Figure 8**). Canonical axis 2 (Can 2) separated C- and H-trees from M-trees, taking into account 6.7% of the variability. No separation was observed along canonical axis 3 (data not shown). Wilk’s λ found significant differences between classes (*F* = 4.03, *P* < 0.05) and the Mahalanobis squared distance between defoliated trees and control showed probabilities lower than 0.05, which indicated that these classes were different from the control (*P* = 0.03 and *P* = 0.005 for M- and H-trees, respectively). The Mahalanobis squared distance between M and H-trees was not significant (*P* = 0.11).

The results of the canonical discriminant analysis showed how the defoliation intensity modified the overall carbon allocation of balsam fir trees (**Table 4**). Can 1 was negatively correlated with the variables representing carbon (leaf starch) and growth (growth rate and number of cells). Thus, the sign of the correlation between the other variables and Can 1 represented either the presence (with a positive correlation) or absence (with a negative correlation) of trade-off. RWC and the variables representing defense (number of monoterpenes and number of sesquiterpenes) were negatively correlated with Can 1 (**Table 4**). A greater defoliation was mainly associated with lower contents or amount of these variables, thus indicating the absence of a trade-off. Only soluble sugars showed positive correlation with Can 1 but with low correlation values (0.26 and 0.17 for leaves and wood, respectively).

**Table 4:** Correlation coefficients between the canonical axes (Can 1 and Can 2) and variables used in the multivariate analysis.

Categories	Variables	Can 1	Can 2
Water	RWC	-0.70	0.11
Carbon	TSS-L	0.26	0.02
	TSS-W	0.17	-0.55
	Sta-L	-0.95	-0.02
	Sta-W	-0.33	-0.34
Growth	Cell.day^-1^	-0.76	-0.18
	*A*	-0.74	-0.06
Defense	β-phe	-0.12	0.59
	δ-3*-*c	-0.46	0.19
	Monoterpenes	-0.70	-0.09
	Sesquiterpenes	-0.72	0.27

*RWC, relative water content of the twigs (%); TSS-L, total soluble sugar in the leaf (mg/g_*dw*_); Sta-L, starch content in the leaf; β-phe, β-phellandrene in the leaf (mg/g_*dw*_); δ-3*-*c, δ-3*-*carene (mg/g_*dw*_) in the leaf; TSS-W, total soluble sugar in the wood (mg/g_*dw*_); Sta-W, starch content in the wood (mg/g_*dw*_), number of monoterpenes or sesquiterpenes.*

Very low correlations were found with Can 2 (**Table 4**), except for sugars and starch in wood (negative correlations) and β-phellandrene (positive correlation). These correlations indicate a lower amount of sugars and starch in M-trees but higher concentration of β-phellandrene, which is in agreement with the values observed in **Table 2**.

## Discussion

### Defense-Growth Trade-off

**FIGURE 9:**
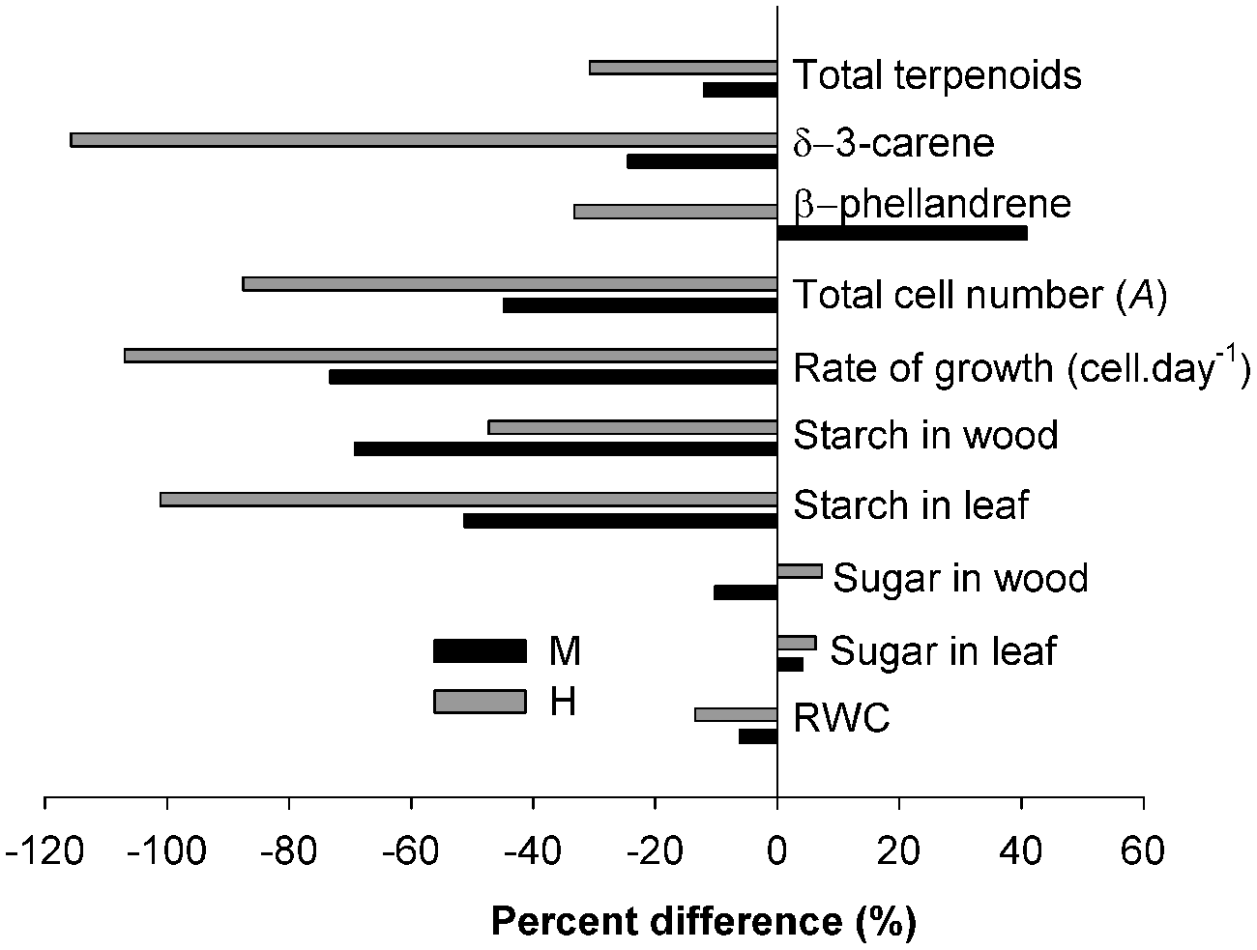
**Percentage difference between values measured in the C-trees and those measured in the M- and H-trees**.

The allocation to both non-structural and structural carbon was highly dependent on the defoliation intensity. In this study, the drastic depletion of the starch reserve in both needles and wood and the severe reduction in wood formation at higher defoliation were thus coherent with the theoretical patterns (**Figure 1**) predicting that increased defoliation will lead to a depletion in carbon and less growth. Expressed as a percentage difference, the reduction in leaf starch and radial growth were both over 100% in H-trees (**Figure 9**). Growth of mature trees is not often limited by carbon availability, and only under defined circumstances, such as severe defoliation, can carbon supply become very limiting (Palacio et al., 2014). During a spruce budworm outbreak, the growing buds are repetitively eaten year after year. Larvae can also break (cut) the young and unlignified twigs. Combined with the loss of older foliage (either naturally or by insect feeding), the defoliation causes a drastic reduction in leaf area. The results of this study show that starch storage was a very strong indicator of the seasonal tree carbon status according to the different intensity of defoliation and could eventually be used to predict long-term tree survival following the outbreak.

Wood formation in defoliated trees gradually reflected the limited plant carbon resources, as non-structural carbohydrates play a crucial role in xylem production (Deslauriers et al., 2014). As a whole, the carbon invested in tree-ring formation (i.e., TRW and cell wall accumulation, **Figure 3**) decreased with increasing defoliation intensity, which is in agreement with the theoretical pattern (**Figure 1A**). The lower cell wall accumulation will directly reduce the structural carbon sequestration in defoliated trees.

The array of terpenoid compounds found during the growing season was indicative of wide constitutive terpenoid defenses (Sampedro, 2014). This could explain why the percentage reduction of terpenoids was lower (less than 40% for both M-and H-trees, **Figure 9**) compared with the percentage of reduction of growth (more than 75%) and storage (more than 50%). Conifers produce terpenoids that are toxic to insects or that negatively affect the physiology of the invading insect or their offspring (Keeling and Bohlmann, 2006). Taking the whole growing season, fewer terpenoid compounds were found at increasing defoliation intensity, indicating, at first sight, no trade-off with growth (**Figure 1B**). Thus, lower allocations in structural and non-structural carbon also correspond to a lower allocation to terpenoid compounds. These results are in agreement with the theory of both the CNB and GDBH (Herms and Mattson, 1992; Koricheva et al., 1998; Sampedro, 2014). Recently, Villari et al. (2014) also observed a positive relationship between growth and terpenoid allocation in Scots pine. However, some specificity in the concentration at a certain period or in the presence/absence of specific compounds could diverge from the absence of trade-off (see Discussion below about terpenoid production).

### Defoliation and Starch Depletion

Our results show that both soluble sugars and starch were less concentrated in wood than in leaves, which is in agreement with the typical pattern in evergreen trees (Hoch et al., 2003; Fajardo et al., 2013). In H-trees, a much lower increase in the starch pool was measured at the end of June. The seasonal starch pattern found in defoliated trees suggests the occurrence of a carbon imbalance (i.e., a quasi-absence of carbon partitioning to reserves) during most of the summer because storage builds-up only when all the other demands have been satisfied (Martinez-Vilalta, 2014). This insufficient carbon storage could eventually compromise the ability of trees to defend themselves and grow (see Production of Defense Components, Wood Formation) as well as to recover and survive (Myers and Kitajima, 2007). In leaves, the typical starch summer accumulation was measured in M- and H-trees, but the amounts were distinctly lower compared to the control. The H-trees stored half as much starch (50 mg/g) than C-trees in leaves. A reduction of 50% of starch reserves in all the compartments (roots, stem, and leaves) was also observed on manually defoliated (50%) *Pinus resinosa* trees with a more pronounced effect on needles (Vanderklein and Reich, 1999). In completely defoliated *Pinus pinaster*, the carbohydrate pools (starch and soluble sugars) decreased by 36% in all organs (Jacquet et al., 2014). Although we did not measure root starch, starch quantity in coarse, and fine roots is known to decrease with increasing levels of defoliation (Webb, 1980; Li et al., 2002) according to the same pattern observed in the other parts of the tree (Vanderklein and Reich, 1999; Kosola et al., 2001). The internal remobilization of starch, either from stem and roots, can contribute to the processes of growth or defense during defoliation, when carbon assimilation is reduced (Chapin et al., 1990).

In addition, we observed that the leaf starch pool was consumed after only 2 weeks in H-trees, while it decreased gradually until DOY 231 in C-trees. This difference of about 60 days showed that trees severely affected by spruce budworm have little ability to store starch, and definitely consume it faster. This strong and fast summer reduction in starch is indicative of very high passive storage (Dietze et al., 2014), with any further acquisition of carbon not partitioned to starch storage in leaves and any additional carbon exported later in the growing season.

In autumn, however, similar rebuilding in the stem starch pool was found between defoliation classes, as also observed for total NSC in completely defoliated *Pinus pinaster* (Jacquet et al., 2014). NSC reserves increase in late-summer, when net primary production exceeds growth demands in order to sustain maintenance respiration during winter (Richardson et al., 2013).

The response of the non-structural available soluble sugars diverges from the starch response. Their amount in defoliated trees was similar to that of non-defoliated trees and the positive correlations in CAN1 for both leaves and wood indicate a possible trade-off (**Figure 1B**), although the correlations were of very low intensity. The percentage change in soluble sugars was also very low (**Figure 9**). In Douglas fir affected by an ascomycete causing the disease called Swiss needle cast, the mean growing season glucose and fructose contents in twigs and needles were unrelated with the functional leaf area, presumably retained in the crown to maintain primary growth there at the expense of the trunk (Saffell et al., 2014). Another possible explanation for the low variation of soluble sugars is their key role in maintaining osmotic functions and metabolism (including growth, respiration, *N* assimilation, and production of defense compounds) which need the coordination of limited mobile carbon resources (Dietze et al., 2014). In this study, lower RWC (8% less overall in H-trees compared with C-trees) was found in the twigs indicating a poorer water status in the twigs of defoliated trees. Therefore, free sugar accumulation for osmotic adjustments under a lower water content could explain the small increase in soluble sugars in defoliated trees.

### Production of Defense Components

From a qualitative point of view, fewer kinds of monoterpenes and sesquiterpenes were formed with an increasing defoliation level (M- and H-trees). Thus, over the entire growing season, many compounds, such as 3-carene, β-myrcene, terpinolene, β-caryophyllene, and β-bisabolene, were found in lower percentages. The biosynthesis of terpenoids is directly dependent on carbon-based compounds: both mono- and sesquiterpene derive from isopentenyl diphosphate (IPP) synthesized in the cytosol of needle tissues by the acetate/mevalonate pathway and in a second way, in plastids by the pyruvate/glyceraldehyde-3-phosphate pathway (Gershenzon et al., 1989; Trapp and Croteau, 2001; Hall et al., 2011). The precursor of monoterpenes, geranyl diphosphate (GPP), derives from the pyruvate/glyceraldehyde-3-phosphate pathway (Trapp and Croteau, 2001). In the case of reduced photosynthesis, C-reserve from sugars and starch will be used to generate the ATP and NADPH required for the synthesis of monoterpenes (Niinemets et al., 2004). Thus, as non-structural carbon was greatly reduced (as starch in the H-trees), we argue that fewer kinds of defense components were formed over the whole growing season because of a deficit in carbon reserves (pattern in **Figure 1A**).

Quantitative changes were observed in the two analyzed compounds, but divergence in tree response (presence and absence), the great variability among single trees concentrations and the variation pattern at the time of larval defoliation did not allow us to accurately determine the theoretical pattern. δ-3-carene was greatly reduced in H-trees (a reduction of more than 100%, **Figure 9**) and in M-trees (25% reduction, **Figure 9**) compared to C-trees. This monoterpene is a well-known defense component against spruce budworm (Bauce et al., 1994; Carisey and Bauce, 1997) that interferes with the normal development of the insect (especially on the sixth-instar development). However, in the defoliated trees that were synthesizing δ-3-carene, the concentration was very high during the period of active defoliation, although it was only found in one tree out of six and three trees out of six in H- and M-trees, respectively. The concentration of β-phellandrene was higher in M-trees compared to H- and C-trees, especially during the defoliation period. This compound is known to be released as a response to injuries when trees are heavily defoliated by spruce budworm (Caron et al., 2013).

Among the other terpenoid compounds found, linalool was intriguing: this monoterpene was present only in defoliated trees (**Figure 5**), suggesting an inductive response to spruce budworm. In Sitka spruce, linalool was uniquely up-regulated as an induced volatile emission by weevil feeding (Miller et al., 2005). The percentage occurrence of camphor, maltol, and myrtenal were also higher in defoliated trees. In this case, the emission of camphor and maltol may be more associated with the larger number of broken resin ducts in needles, because insect damage led to a higher release of volatile compounds in needle tissues (Holopainen and Gershenzon, 2010) and was not associated with a defense mechanism.

According to Herms and Mattson (1992), the cost of defense will vary over the course of the growing season. In this study, high levels of δ-3-carene and β-phellandrene were synthesized late in the season. Therefore, as growth was slowing down, allocation to other processes, such as defense and storage, may occur to eventually reduce trade-offs at a time of high demand such as during defoliation.

### Wood Formation

Current-year assimilates rather than stored carbon are mostly used to build xylem (Hansen and Beck, 1994; Carbone et al., 2013) with starch conversion in the phloem being used only at the beginning of wood formation (Begum et al., 2013). After seasonal build-up of starch reserves, a certain amount of this stored carbon is withdrawn to support cambial activity and shoot growth (Fatichi et al., 2014), which was not possible for H-trees. Moreover, during the period of intense defoliation (end of June, DOY 160–180), wood formation in H-trees temporarily ceased as 100% of the new growing buds were eaten (data not shown), whereas M- and C-trees had their exponential growth phase, suggesting a carbon deficit for wood formation. The rate of cell division was therefore lower in defoliated trees, resulting in fewer xylem cells produced along the radius. A reduction in cell number, especially latewood, is the first impact of a spruce budworm outbreak (Krause and Morin, 1995b). However, the trends of secondary wall formation versus cell production were similar between control and defoliated trees.

## Conclusion

This study analyzed the effect of defoliation to test the presence of a trade-off between allocation to growth, carbon storage, and defense. The investigation was realized on adult trees in the field in order to compare plants naturally defoliated for several years. However, such a monitoring prevented to profit from a more comprehensive experiment with completely randomly selected individuals based on replicates. This condition limited the strength of the statistics and their generalization, although the different analyses employed in this study converged toward similar results.

The findings did not support a trade-off between growth and defense. Carbon allocation to defense and growth is expensive because terpenoids are compounds rich in carbon and cell wall formation require a lot of sucrose. Thus, the heavily defoliated trees exhibited less diversity of defense compounds. This strategy of saving carbon could be an advantage only if production focuses on fewer but more effective compounds (Herms and Mattson, 1992). A carbon saving strategy was effectively used for wood formation by the production of fewer tracheids. We found that the starch amount was most indicative of tree carbon status occurring during a spruce budworm outbreak, so future research should focus on the mechanism of starch utilization for survival and growth following an outbreak. Moreover, a whole-tree carbon allocation and remobilisation, including leaf, twig, wood, and root storage, could help to better define the dynamics of recovery and growth.

## Conflict of Interest Statement

The authors declare that the research was conducted in the absence of any commercial or financial relationships that could be construed as a potential conflict of interest.
